# Work Engagement, Burnout, and Self-Care Index Scores in Relation to Self-Reported In-Role and Extra-Role Job Performance Among Nursing Personnel in Six Public Hospitals in Northern Greece: A Secondary Analysis of a Multicentre Cross-Sectional Survey

**DOI:** 10.3390/healthcare14203460

**Published:** 2026-10-15

**Authors:** Thalia Bellali, Chrysovalantis Karagkounis, Evlabia Kostelidou, Polyxeni Liamopoulou, Georgios Manomenidis

**Affiliations:** 1 School of Social Sciences, Postgraduate Course–Health Care Management, Hellenic Open University, 26335 Patras, Greece; 2 Department of Nursing, International Hellenic University, 57001 Thessaloniki, Greece; tzeniliam@gmail.com; 3 Department of Care for the Elderly, Directorate of Social Policy and Public Health, Municipality of Katerini, 60133 Katerini, Greece; kchrysov@gmail.com; 4 Papageorgiou General Hospital, 57001 Thessaloniki, Greece; evikostelidou@yahoo.gr; 5 Department of Nursing, Democritus University of Thrace, 68100 Alexandroupolis, Greece; gmanomen@nurs.duth.gr

**Keywords:** work engagement, burnout, self-care, job performance, nursing personnel, healthcare workforce management, secondary analysis

## Abstract

**Highlights:**

**What are the main findings?**

• In a pandemic-era hospital sample, work engagement had the numerically largest standardized coefficients for self-reported in-role and extra-role performance after simultaneous adjustment.
• Higher scores on the study-developed Self-Care Index showed exploratory positive associations with both performance scores; burnout showed no statistically detectable mutually adjusted association.

**What are the implications of the main findings?**

• Limited construct-validity evidence for the Self-Care Index requires an exploratory interpretation; the study does not establish causal effects or intervention effectiveness.
• Prospective studies should use validated measures, independently assessed performance, and objective organizational and patient outcomes to evaluate potential management strategies.

**Abstract:**

**Background/Objectives:** This study examined mutually adjusted associations of work engagement, burnout, and Self-Care Index scores with self-reported in-role and extra-role performance among nursing personnel. **Methods:** This secondary analysis used pandemic-era cross-sectional data from 586 nurses and nurse assistants recruited by convenience sampling in six public hospitals in Northern Greece (December 2021–April 2022). Measures included the Utrecht Work Engagement Scale–9, Oldenburg Burnout Inventory, a study-developed 35-item Self-Care Index, and a six-item job-performance adaptation. Confirmatory factor analysis (CFA) included 579 participants. Three complete-case regression models (*n* = 574) simultaneously included the three focal scores, gender, age, and managerial responsibility. Sensitivity analyses used HC3 standard errors and hospital fixed effects; coefficient *p*-values were nominal. **Results:** CFA provided only partial support for distinguishing the performance dimensions, with substantial factor overlap (r = 0.833) and residual misfit (root mean square error of approximation = 0.092). Performance scores showed upper-end concentration. Work engagement had the numerically largest standardized coefficients for in-role (β = 0.285) and extra-role performance (β = 0.402; both *p* < 0.001). Self-Care Index scores showed positive associations (β = 0.193, *p* < 0.001; β = 0.100, *p* = 0.018, respectively). Burnout showed no statistically detectable mutually adjusted association. The complementary total-score model showed a similar pattern. **Conclusions:** Engagement was positively associated with self-reported performance. Self-Care Index findings remain exploratory because construct validity is not established. Shared self-report measurement and the cross-sectional design preclude causal or intervention-effectiveness conclusions. Prospective evaluation using validated measures and independently assessed performance is needed.

## 1. Introduction

Healthcare systems facing workforce shortages and constrained resources depend not only on workforce capacity but also on organizational conditions that support reliable staff functioning and performance [1]. Within hospital services, nursing personnel’s job performance is a management-relevant workforce outcome [2]. Staff engagement has been associated with patient safety outcomes [3], while nurse burnout has been associated with poorer patient safety, satisfaction, and quality of care [4]. Favorable nursing practice environments have been associated with lower burnout and higher work engagement [5]. More broadly, unsafe care continues to impose substantial human and economic costs on healthcare systems [6,7]. These links provide an important management context for studying nursing performance, although the present study did not directly assess patient safety, service efficiency, costs, or patient outcomes.

Job performance is multidimensional. In-role, or task, performance refers to behaviors required to fulfill formal job responsibilities effectively, whereas extra-role, or contextual, performance encompasses discretionary behaviors that support the broader social and organizational environment in which core tasks are performed [2,8]. Extra-role/contextual performance overlaps conceptually with organizational citizenship behavior (OCB), including voluntary helping and cooperative behaviors, but the constructs should not be treated as completely synonymous [8,9]. Distinguishing in-role from extra-role performance may therefore provide greater insight than an overall performance score alone when examining how workforce-related factors relate to different aspects of employees’ self-reported work functioning.

Work engagement, burnout, and self-care represent related but non-equivalent workforce constructs that may be relevant to performance. Work engagement is characterized by vigor, dedication, and absorption and has a well-established positive association with job performance, including task and contextual performance [10,11,12]. Burnout reflects adverse work-related experiences and has been associated with impaired occupational functioning and poorer job performance among nurses [13]. Self-care broadly encompasses behaviors through which healthcare professionals attempt to preserve their physical and psychological well-being and manage occupational demands [14]. Evidence directly linking self-care behaviors to job performance remains comparatively limited. However, a recent study of Greek critical-care nursing personnel found that higher self-care was positively associated with job performance [15].

Demographic and occupational characteristics may also be associated with self-reported work performance, although their relationships vary across settings. Previous nursing studies have reported differences in job performance according to characteristics such as gender, age, educational level, and professional experience. However, the direction and magnitude of these associations are not consistent across populations [15,16]. In Greece, nursing personnel have reported substantial fatigue and burnout during the COVID-19 pandemic [17]. At the same time, job satisfaction has also been examined among Greek healthcare professionals [18], and post-pandemic evidence has identified comparatively greater burnout and lower job satisfaction among nurses than among other healthcare workers [19]. Leadership and the nursing practice environment are associated with engagement and burnout, underscoring the importance of interpreting individual workforce outcomes within their organizational context [5,20].

The source dataset used in the present analysis was originally collected during the COVID-19 pandemic for an MSc research project among nursing personnel from six public hospitals in Northern Greece. It was previously described in the corresponding MSc thesis [21]. That work primarily reported descriptive and bivariate relationships among workforce well-being and performance variables. A related 2025 study in Greek critical-care nursing personnel also reported an association between self-care and job performance [15]. The present study addresses a distinct analytical question by simultaneously examining work engagement, burnout, and Self-Care Index scores within a common multivariable framework; estimating separate models for self-reported in-role and extra-role performance; retaining total job performance as a complementary overall summary; and evaluating the stability of the principal associations using HC3 heteroscedasticity-consistent and hospital fixed-effects sensitivity analyses.

Accordingly, the primary objective was to examine the mutually adjusted associations of work engagement, burnout, and Self-Care Index scores with self-reported in-role and extra-role job performance among nursing personnel in six public hospitals in Northern Greece. Total job performance was examined as a complementary overall outcome. Gender, age, and managerial responsibility were included as common adjustment variables, while additional demographic and occupational characteristics were examined exploratorily. We hypothesized that higher work engagement and higher Self-Care Index scores would be associated with higher in-role and extra-role performance. In contrast, higher burnout would be associated with lower performance. Given the cross-sectional design, these hypotheses address associations, not causal effects.

## 2. Materials and Methods

### 2.1. Study Design, Data Source, and Setting

The present study is a secondary analysis of data from a multicentre cross-sectional survey originally conducted among nursing personnel employed in six public hospitals in Northern Greece. The source dataset was collected for an MSc research project and subsequently deposited as an MSc thesis at the Hellenic Open University [21]. The original data collection occurred from December 2021 to April 2022, following ethical approval granted on 26 November 2021. No new participant recruitment or data collection was undertaken for the present analysis; the current analytical framework, statistical reanalysis, interpretation, and manuscript preparation were undertaken in 2025–2026.

The original survey included two tertiary and four secondary public hospitals in Northern Greece. Recruitment took place in eligible clinical areas across the participating hospitals using non-probability convenience sampling. Hospitals and participants were not selected through probability-based or hospital-stratified sampling, and the source sample was not intended to be nationally representative of the Greek nursing workforce. Supplementary Table S1 presents the six participating hospitals and their classification as secondary or tertiary institutions. Questionnaire distribution followed the same paper-based procedure across participating hospitals.

### 2.2. Data Collection

A convenience sample of nursing personnel employed in the participating hospitals was invited to participate. Eligible participants were registered nurses or nurse assistants holding permanent positions or open-ended contracts and having at least one year of professional experience. Nursing personnel employed under short-term, individual, auxiliary, or other temporary contractual arrangements, personnel on extended leave, and questionnaires lacking valid consent or containing unusable data were excluded. These criteria were intended to ensure that participants had at least one year of exposure to their professional role and workplace context. In the Greek healthcare system, nurse assistants constitute a distinct occupational category with nursing-assistant qualifications obtained through secondary or post-secondary vocational education. Their role includes basic patient care and delegated nursing activities, such as personal hygiene assistance and vital-sign measurement, under the direction and responsibility of registered nurses [22]. In the present survey, participants self-reported their professional role through a separate questionnaire item that distinguished nurses from nurse assistants; educational attainment was recorded separately.

Data were collected using anonymous, paper-based, self-administered questionnaires. Each questionnaire was accompanied by written information describing the study purpose, voluntary participation, confidentiality, and the consent process. Participants could decline participation or withdraw before returning the anonymous questionnaire. Because responses were anonymous, participants could not withdraw individual questionnaires after submission. Immediate completion was often impractical because of workload and COVID-19 infection-control requirements; consequently, completed questionnaires were collected during regular visits to the participating hospitals, generally at least weekly. No electronic survey links or parallel online data-collection channels were used.

Because questionnaires were anonymous, identity-based verification of unique individual participation was not possible. As a data-quality check, the analytical database was screened for repeated record identifiers and exact duplicate response records; none were identified among the 586 questionnaires included. This procedure could identify duplicate records but could not establish participant identity.

A total of 817 paper questionnaires were distributed across the six hospitals; 586 were returned and retained in the descriptive analytical dataset, and 231 were not returned. No returned questionnaire was excluded for absent consent or classified as wholly unusable. The overall questionnaire return rate was therefore 71.7% (586/817). Hospital-specific questionnaire-distribution denominators, the total number of eligible personnel approached, and refusal counts were not retained; consequently, hospital-specific return rates and a population-based participation rate cannot be calculated. The 71.7% figure should therefore be interpreted strictly as an overall questionnaire return rate.

### 2.3. Study Size and Analytical Sample

Because this study is a secondary analysis of an existing dataset, the available sample size was determined by the original survey rather than by a new recruitment target for the current analysis. The source dataset comprised 586 returned questionnaires retained for descriptive analysis. For the present multivariable analyses, 574 participants had complete valid data for the performance outcomes and all six variables included in the common adjustment set. They were therefore included in each regression model. No retrospective observed-power calculation was used to judge the adequacy of the present analysis; statistical precision is reported using coefficient estimates and 95% confidence intervals.

### 2.4. Data Collection Tools

The study collection tool comprised five parts: (a) demographic and occupational characteristics, (b) the Utrecht Work Engagement Scale (UWES), (c) the Oldenburg Burnout Inventory (OLBI), (d) the Self-Care Index, and (e) the Job Performance Scale (JP).

#### 2.4.1. Demographic and Occupational Characteristics

Demographic and occupational characteristics were assessed using a questionnaire developed for the present study. Variables included gender, age, marital status, educational level, professional role (registered nurse or nurse assistant), years of professional experience, managerial responsibility, hospital type (secondary or tertiary), and clinical department. For analysis, clinical departments were grouped into Ward-Based Inpatient Units and critical/interventional units according to the predominant clinical function and care-delivery profile of each setting. Ward-Based Inpatient Units included medical, surgical, pediatric, psychiatric, obstetric–gynecological, and other specialty inpatient wards providing continuous ward-based care. Critical/interventional units included emergency, intensive, and coronary care; operating theatre and anesthesia services; dialysis units; and other acute or procedure-oriented clinical settings. Department entries referring to administrative, support, cross-cutting, or insufficiently specified placements were not included in the department-specific comparison. The classification was applied consistently to the original department labels before repeating the analyses.

#### 2.4.2. Utrecht Work Engagement Scale (UWES)

Work engagement was conceptualized according to the established dimensions of vigor, dedication, and absorption [23,24]. The present study used the nine-item short form of the Utrecht Work Engagement Scale (UWES-9) [23], administered using Greek wording derived from the previously examined Greek UWES. Greek evidence has supported the three-factor structure of the UWES and its measurement invariance relative to a Dutch sample. However, that study should not be interpreted as a separate formal validation of the Greek UWES-9 short form [25]. The UWES-9 contains three items each for vigor, dedication, and absorption, rated from 0 (“never”) to 6 (“always/every day”). The overall score was calculated as the mean of the nine items and the three dimension scores as the means of their respective items, subject to the completion rules described in Section 2.6. Higher scores indicate greater work engagement. Reporting both the overall score and the three subscale scores was supported by the correlated three-dimensional structure described for the UWES-9 and by the instrument’s scoring guidance. The previously examined Greek-language items were administered without additional translation, wording modification, or cultural adaptation for the present study. The UWES-9 was used under the conditions specified by its developer for non-commercial academic research. Internal consistency was evaluated in the present sample for the overall scale and each of its three dimensions.

#### 2.4.3. Oldenburg Burnout Inventory (OLBI)

Burnout was assessed using the Greek-language version of the 16-item Oldenburg Burnout Inventory (OLBI) examined in Greek occupational samples by Demerouti et al. [26]. The OLBI comprises two eight-item dimensions: exhaustion and disengagement from work. Each dimension contains positively and negatively worded items. Responses are provided on a four-point Likert scale ranging from 1 (“strongly agree”) to 4 (“strongly disagree”). After verifying the administered Greek questionnaire and its item orientation, items 2, 3, 4, 6, 8, 9, 11, and 12 were reverse-scored using the transformation 5 − x so that higher scores consistently indicated greater exhaustion, disengagement, and overall burnout. Disengagement was calculated from items 1, 3, 6, 7, 9, 11, 13, and 15, whereas exhaustion was calculated from items 2, 4, 5, 8, 10, 12, 14, and 16. The two subscale scores were calculated as the means of their respective eight items, and the overall OLBI score was calculated as the mean of all 16 items.

The established two-dimensional structure of the OLBI supported separate reporting of exhaustion and disengagement. The overall 16-item mean was additionally used as a summary burnout indicator in the multivariable analyses and should not be interpreted as evidence of a separate higher-order factor. The previously examined Greek-language items were administered without additional translation, wording modification, or cultural adaptation for the present study. The OLBI was used and cited in accordance with its published academic source. Internal consistency was evaluated in the present sample for the overall scale and both dimensions.

#### 2.4.4. Study-Developed Self-Care Index

Self-care-related behaviors and experiences were assessed using a Greek-language questionnaire included in the original MSc survey [21], described here as a 35-item study-developed Self-Care Index. Related self-care assessment has also been reported in Greek nursing research [27]. The source study documentation identified the health-behavior literature, including the work of Kulbok et al. [28], and the Institute for Functional Medicine Self-Care Questionnaire as conceptual resources. The accessible Version 2 of the latter is cited as a content reference [29]; the exact IFM edition consulted during the original development process could not be verified from the available study records.

The source documentation described four intended content areas: physical well-being, emotional/spiritual well-being, professional life, and social life [21]. The archived items cover health-related practices, including eating, hydration, exercise, sleep, relaxation, and consumption habits; emotional experiences and self-directed attitudes, including meaning, optimism, and self-kindness; work-related experiences, including enjoyment of work, perceived ability to manage challenges, and boundary setting; and social resources and relationships, including family support, companionship, and help-seeking. These areas describe the breadth of item content and should not be interpreted as empirically established subscales.

The available records did not provide a reproducible account of the initial item pool, item generation, selection, reduction, or linguistic adaptation. Consequently, the historical rationale for selecting exactly 35 items could not be reconstructed. Documentation of formal expert-panel content validation, cognitive interviewing, or separate linguistic pilot testing was also unavailable. No comprehensive evidence of structural, convergent, discriminant, or criterion validity was available for the present 35-item composite. It should therefore be regarded as an exploratory multidomain composite, without established equivalence to a validated Greek version of either source instrument.

Verification against the archived final questionnaire and source dataset confirmed 35 items, corresponding to questionnaire items 48–82. Although the MSc thesis described 34 items and presented 34 item-level frequency distributions [21], the final questionnaire and dataset also contained an item concerning the perceived ability to ask for help. All 35 archived items were retained in the present reanalysis. Brief item-content descriptors, questionnaire numbering, dataset mapping, and operational scoring are provided in Supplementary Table S8.

Responses were coded from 1 (“never”) to 6 (“always”). Values outside the permitted response range were treated as missing before scoring. Items 2 (fast-food consumption), 7 (alcohol consumption), 8 (smoking), and 9 (coffee consumption) were reverse-coded as 7 − x; the remaining 31 items retained their original coding. The overall score was calculated as the arithmetic mean of valid, directionally coded responses when at least 28 of the 35 items were available, using the number of valid responses as the participant-specific denominator. Otherwise, the score was coded as missing. This 80% minimum-completion requirement was an operational rule adopted for the present reanalysis, rather than a prospectively prespecified or validated instrument-specific rule. No missing responses were imputed.

A single overall mean was retained to examine whether a broad pattern of self-care-related responses was associated with self-reported job performance. Retaining the complete archived item set avoided selecting items according to their associations with the study outcomes. Equal weighting of available items provided a transparent descriptive aggregation rule and preserved the 1–6 response metric. This approach does not assign equal weight to conceptual domains, and different item-response profiles may produce the same overall score. The resulting composite was not interpreted as an estimate of a single latent trait. No domain-specific scores were calculated because the proposed structure had not been independently confirmed. Higher scores indicate a more favorable response pattern according to the operational scoring scheme. Cronbach’s alpha was reported descriptively and was not used to justify aggregation or establish construct validity. Associations involving this composite were interpreted as exploratory.

#### 2.4.5. Self-Reported Job Performance

Self-reported job performance was assessed using a six-item Greek-language adaptation of selected items originating from the broader task/contextual performance framework reported by Goodman and Svyantek [30]. The six-item adaptation had previously been used in Greek academic research [31] and was administered in the source survey without further wording modification. Detailed documentation of a formal forward–backward translation, expert-panel review, cognitive interviewing, or separate linguistic pilot-testing procedure was not available. The measure should therefore be regarded as a previously used Greek-language adaptation rather than as a formally validated standalone Greek instrument. Three items were designated as in-role items (items 2, 3, and 5) and three as extra-role items (items 1, 4, and 6). Participants were instructed to answer with reference to the preceding four months. Responses ranged from 0 (“does not describe me at all”) to 6 (“describes me perfectly”). In-role and extra-role scores were calculated as the means of their respective three items, subject to the completion rules described in Section 2.6. The total score was calculated directly as the mean of valid responses across the six items and retained as a complementary overall summary; it was not treated as a psychometrically distinct third dimension. The two domains contributed equally when all six items were observed. When an item was missing, their contributions reflected the number of valid items rather than equal weighting of the two domain means. Supplementary Table S9 provides brief item-content descriptors, questionnaire numbering, dataset mapping, allocation to the in-role and extra-role scores, and the operational scoring procedures.

Given the absence of prior structural validation of this six-item Greek-language adaptation, its dimensional structure was additionally evaluated in the present reanalysis by comparing a one-factor model with a correlated two-factor model representing in-role and extra-role performance (Section 2.6). This analysis was intended to provide sample-specific evidence regarding the proposed score structure and was not regarded as independent validation of two clearly distinct performance constructs. Internal-consistency coefficients were also calculated for the total score and both three-item scores.

#### 2.4.6. Instrument Use

The UWES was used for non-commercial academic research in accordance with the applicable conditions specified by its developer. The OLBI was used and cited according to its published academic sources. The six-item job-performance measure represented a previously used Greek-language academic adaptation. In contrast, the Self-Care Index was a study-developed Greek-language composite informed by the health-behavior and self-care literature. Its documented conceptual sources and the limitations of the available development records are described in Section 2.4.4. Supplementary Tables S8 and S9 provide author-prepared content descriptors, questionnaire-to-dataset mappings, and operational scoring procedures for the Self-Care Index and the six-item job-performance adaptation, respectively. Full item wording is not reproduced because authorization for its public reproduction could not be verified from the available records. The descriptors are provided for methodological documentation and should not be used as substitute questionnaire items.

### 2.5. Ethical Considerations

The original cross-sectional survey was conducted in accordance with the Declaration of Helsinki and received approval from the Institutional Review Board of the 3rd Regional Health Authority of Macedonia (protocol code Δ3β/61972; approval date 26 November 2021) before participant recruitment and data collection. The original data were collected from December 2021 to April 2022. All participants received written study information and provided informed consent before completing the anonymous questionnaire. No new participant recruitment or data collection was undertaken for the present secondary analysis.

### 2.6. Data Analysis

Statistical analyses were performed using IBM SPSS Statistics for Windows, version 26.0 (IBM Corp., Armonk, NY, USA), with selected regression and sensitivity analyses independently reproduced in Python as described below. For the present reanalysis, the source dataset was screened for completeness, values outside permitted response ranges, repeated record identifiers, and exact duplicate response records. Values that could not be verified as valid responses were treated as missing; no model-based or single-value imputation was performed.

To apply scoring consistently in the present reanalysis, operational minimum-completion thresholds were applied as follows: at least seven of nine UWES-9 items for the overall score and at least two of three items for each UWES dimension; at least 13 of 16 OLBI items for the overall score and six of eight items for each OLBI dimension; at least five of six job-performance items for the complementary total score and two of three items for each in-role and extra-role score; and at least 28 of 35 Self-Care Index items. When the relevant threshold was not met, the corresponding score was coded as missing. These minimum-completion thresholds were operational scoring rules applied during the present reanalysis to avoid calculating scores from substantially incomplete item sets; they were not prospectively prespecified or formally validated instrument-specific missing-item rules. No model-based or single-value imputation was performed.

The dimensional structure of the six-item job-performance adaptation was evaluated using confirmatory factor analysis of the six ordered-response items. A one-factor model was compared with a correlated two-factor model specifying the three in-role items and three extra-role items as separate factors. The CFA was conducted in JASP (Version 0.17) using the SEM/CFA module (lavaan engine) and was estimated using diagonally weighted least squares (DWLS) based on polychoric correlations to account for the ordered categorical response format. The CFA analytic sample comprised 579 participants with complete responses to all six job-performance items. Seven participants had incomplete item-level job-performance data (five had one missing item and two had all six items missing) and were excluded from the CFA; no imputation was performed. Model evaluation included standardized factor loadings, the comparative fit index (CFI), Tucker–Lewis index (TLI), root mean square error of approximation (RMSEA) with its 90% confidence interval, and standardized root mean square residual (SRMR). The factor correlation was also reported. Supplementary Table S2 provides detailed results.

Descriptive and bivariate analyses used all scores meeting these completion requirements, whereas each multivariable model included participants with complete data for its outcome and all included predictors. Cronbach’s alpha coefficients were calculated using participants with complete responses to all constituent items of the corresponding scale or subscale. The complete-case analytic sample size was reported for each regression model; all three models included the same 574 participants. Categorical variables are presented as frequencies and percentages, and continuous variables as means and standard deviations. For all study scales and subscales, valid sample sizes, observed minimum and maximum values, and sample skewness were calculated to characterize their empirical distributions. For the three bounded job-performance outcomes, potential upper-end concentration was additionally described using both the proportion of participants attaining the maximum possible mean score of 6 and the proportion scoring within one scale point of the upper bound (mean score ≥ 5.0). The latter was used as a descriptive indicator rather than as a formal diagnostic threshold for a ceiling effect. Because boundedness and outcome skewness do not by themselves invalidate linear regression, the appropriateness of the fitted models was evaluated primarily from the residual-versus-predicted plots, normal Q–Q plots, standardized residuals, and influence diagnostics. HC3 heteroscedasticity-consistent standard errors were used as a sensitivity analysis.

Exploratory bivariate analyses were used to describe unadjusted relationships between participant characteristics and the three job-performance scores. Two-category group comparisons used the Mann–Whitney U test, comparisons involving more than two categories used the Kruskal–Wallis H test, and associations between continuous variables were assessed using Spearman’s rank correlation coefficient (ρ). These analyses were exploratory; their *p*-values were not adjusted for multiple comparisons and were not used to select variables for the multivariable models. Full exploratory results are presented in Supplementary Table S3.

Three multiple linear regression models were fitted for in-role performance, extra-role performance, and the complementary total job-performance score. All three models used the same six predictors and the same complete-case sample of 574 participants, allowing descriptive comparison of associations across outcomes under a consistent adjustment scheme. Work engagement, burnout, and Self-Care Index scores were the three focal psychosocial variables. Their simultaneous inclusion allowed each association to be estimated conditional on the other two scores. Gender and age provided basic demographic adjustment, while managerial responsibility represented an occupational characteristic related to differences in work responsibilities and role expectations. All six variables were entered simultaneously; selection was not based on exploratory bivariate *p*-values.

The common model was intentionally parsimonious, retaining the three focal psychosocial variables and three demographic or occupational adjustment variables to maintain a consistent specification across outcomes. Educational level, professional role, hospital level, and clinical department were examined exploratorily but were not included in the primary adjustment set. Their omission should not be interpreted as evidence that these characteristics are unrelated to performance or cannot contribute to residual confounding. Professional experience was not entered alongside age because the two variables were strongly correlated (ρ = 0.868, *p* < 0.001); age was retained to avoid redundant information in the common model. Between-hospital differences were examined separately in the hospital fixed-effects sensitivity analyses described below. The fitted models estimate mutually adjusted cross-sectional associations and were not intended to identify causal effects or a definitive set of confounders.

Categorical predictors were dummy-coded, and the reference category for each categorical predictor is specified in the table in Section 3.5. Unstandardized regression coefficients (B), standard errors, 95% confidence intervals, t-statistics, *p*-values, and standardized coefficients (β) were reported. Model performance was evaluated using the F statistic, R^2^, and adjusted R^2^. Comparisons of standardized coefficients within and across outcome models were descriptive; no formal tests of differences between coefficients were performed. The reported coefficient *p*-values assess associations against the null value of zero and do not test whether coefficients differ from one another.

Because participants were drawn from six hospitals with unequal sample contributions, sensitivity to hospital-level differences was assessed in two ways. First, unconditional one-way random-effects models were used to estimate the intraclass correlation coefficient (ICC) for each performance outcome. Second, the three primary regression models were re-estimated with hospital included as a fixed categorical term represented by five indicator variables. The joint contribution of the hospital indicators was evaluated using a partial F test. These analyses were intended to assess sensitivity to between-hospital differences and do not eliminate every possible form of within-hospital dependence. Conventional large-sample cluster-robust standard errors were not used because only six hospital clusters were available.

Multicollinearity was evaluated using variance inflation factors (VIFs) and tolerance values. Regression diagnostics were examined separately for each model. Linearity and variance patterns were evaluated using residual-versus-predicted plots, and residual distribution was evaluated using normal Q–Q plots. Potentially influential observations were examined using Cook’s distance and standardized residuals. Cook’s distance greater than 1 was treated as an indicator of potentially substantial overall influence; 4/(n − k − 1) was used only as a sensitive case-screening threshold and not as an automatic exclusion criterion. Observations with absolute standardized residuals greater than 3 were examined but were not removed solely on this basis. As a sensitivity analysis, all three models were re-estimated using the heteroscedasticity-consistent covariance matrix estimator type 3 (HC3). The primary OLS analyses were performed using IBM SPSS Statistics for Windows, version 26.0 (IBM Corp., Armonk, NY, USA). The regression models, influence diagnostics, and HC3 covariance matrices were independently reproduced in Python version 3.12.13 using a custom matrix-based implementation with NumPy version 2.3.5 and SciPy version 1.17.0. For HC3 estimation, each squared residual contribution was adjusted by (1 − h_ii_)^−2^, where h_ii_ denotes the corresponding leverage value. Full HC3 coefficient estimates, robust standard errors, 95% confidence intervals, test statistics, and *p*-values are presented in Supplementary Table S4. All statistical tests were two-sided. No multiplicity-adjustment procedure was prespecified for the three parallel multivariable outcome models; therefore, the reported coefficient *p*-values are nominal and should be interpreted together with effect estimates, 95% confidence intervals, consistency across outcomes, and the sensitivity analyses. For individual coefficients, *p* < 0.05 was used as the nominal significance threshold.

### 2.7. Use of Generative Artificial Intelligence and Language-Editing Tools

During manuscript preparation and revision in 2025–2026, the authors used ChatGPT (OpenAI) to assist with scientific-language restructuring, consistency checking, and drafting and refining revised text, including the wording used to present and discuss the reported findings. Because the manuscript was developed and revised over an extended period and the authors did not retain a complete model-level usage log, a single historical ChatGPT model/version cannot be identified reliably for all uses and is therefore not retrospectively assigned. Grammarly was additionally used for English-language editing, including grammar, spelling, punctuation, clarity, and stylistic consistency. Neither tool was used for the original data collection, data generation, or the primary statistical analyses. All AI-assisted and language-edited content, suggested revisions, references, and interpretive wording were critically reviewed, independently verified where appropriate, and edited by the authors, who retain full responsibility for the scientific content, accuracy, and integrity of the manuscript.

## 3. Results

### 3.1. Participant Characteristics

The final sample comprised 586 nursing personnel, including 430 registered nurses (73.4%) and 156 nurse assistants (26.6%). Most participants were women (*n* = 477, 81.4%). The mean age was 44.45 years (SD = 9.03), and the mean duration of professional experience was 18.77 years (SD = 9.59). Among the 584 participants with valid marital-status data, 380 (65.1%) were married, while 204 (34.9%) were single, divorced, or widowed. Most participants worked in tertiary hospitals (63.7%), and 69 (11.8%) held managerial responsibility. Participant characteristics are presented in Table 1. Participants were unequally distributed across the six study hospitals, with hospital-specific contributions ranging from 39 to 246 participants. Supplementary Table S1 presents the number and percentage of questionnaires returned from each hospital. Hospital-specific questionnaire-distribution denominators were not available; therefore, hospital-specific questionnaire return rates could not be calculated.

Missingness was limited but varied across variables. Valid data were available for all 586 participants for gender, professional role, educational level, hospital level, and managerial responsibility; for 580 participants for age; for 584 for marital status; and for 577 for professional experience. Of the six age values not included in the age analyses, five were missing and one value of 0 years was treated as invalid. Clinical-department information was missing for 27 participants, and 21 additional recorded placements could not be classified into either department group, leaving 538 participants for department-group analyses. After application of the scale-completion rules used for the present reanalysis, valid scores were available for 583 participants for the UWES-9, 586 for the OLBI, 585 for the Self-Care Index, and 584 for each job-performance outcome. Table 1, Table 2 and Table 3 report valid sample sizes, while Supplementary Table S5 details variable-level missingness and the derivation of the complete-case regression sample.

**Table 1:** Demographic and occupational characteristics of the participants.

Variables	Valid *n*	*n* (%) or Mean (SD)
Gender	586	
Female	—	477 (81.4)
Male	—	109 (18.6)
Age, years	580	44.45 (9.03)
Marital status	584	
Married	—	380 (65.1)
Other (single, divorced, or widowed)	—	204 (34.9)
Educational level	586	
Secondary education	—	155 (26.5)
Tertiary education (TE/PE)	—	281 (48.0)
MSc/PhD	—	150 (25.6)
Professional role	586	
Registered nurse	—	430 (73.4)
Nurse assistant	—	156 (26.6)
Professional experience, years	577	18.77 (9.59)
Hospital level	586	
Secondary	—	213 (36.3)
Tertiary	—	373 (63.7)
Managerial responsibility	586	
Yes	—	69 (11.8)
No	—	517 (88.2)
Clinical department group	538	
Ward-Based Inpatient Units	—	245 (45.5)
Critical/Interventional Units	—	293 (54.5)

Note: Values are presented as *n* (%) for categorical variables and mean (SD) for continuous variables. Valid sample sizes are reported for each variable. Five age values were missing, and one recorded age of 0 years was treated as invalid, resulting in a valid *n* of 580. Marital-status data were missing for two participants, and professional-experience data were missing for nine participants. Clinical department information was missing for 27 participants. A further 21 participants had recorded administrative, support, cross-cutting, or insufficiently specified placements that could not be classified into either of the two clinical-department groups. Consequently, the clinical-department-group analysis included 538 participants. Percentages were calculated using the corresponding valid denominator and may not total 100% because of rounding.

### 3.2. Descriptive Scores and Reliability

Descriptive statistics and internal-consistency coefficients are presented in Table 2. The mean overall work-engagement score was 3.33 (SD = 1.29), and the mean burnout score was 2.53 (SD = 0.48). Following reverse-scoring of the four adversely oriented items, exclusion of values outside the permitted response range, and application of the minimum item-completion requirement, the mean Self-Care Index score was 3.80 (SD = 0.63; *n* = 585). The mean total job-performance score was 4.63 (SD = 0.96); mean in-role and extra-role performance scores were 4.58 (SD = 1.03) and 4.69 (SD = 1.06), respectively. All three job-performance scores covered the full possible range from 0 to 6 and showed moderate negative skewness (total: −1.018; in-role: −0.965; extra-role: −0.966), indicating concentration toward the upper end of the scale. The maximum possible mean score of 6 was attained by 31 participants (5.3%) for total performance, 60 (10.3%) for in-role performance, and 90 (15.4%) for extra-role performance. Moreover, 261 participants (44.7%), 290 (49.7%), and 296 (50.7%), respectively, had mean scores of at least 5.0. These findings indicate appreciable upper-end concentration, particularly for extra-role performance, and suggest possible ceiling-related compression of outcome variability. Internal-consistency coefficients are reported in Table 2. These coefficients describe score consistency within the present sample. They should not be interpreted as evidence of dimensional or construct validity, particularly for the study-developed Self-Care Index and the adapted job-performance measure.

**Table 2:** Descriptive statistics and internal consistency of the study measures.

Scale	*n*	Mean	SD	Observed Range	Skewness	Cronbach’s α
UWES-9 total	583	3.33	1.29	0–6	0.171	0.949
Vigor	583	3.21	1.38	0–6	0.219	0.905
Dedication	583	3.68	1.39	0–6	−0.126	0.876
Absorption	583	3.11	1.38	0–6	0.248	0.864
OLBI total	586	2.53	0.48	1.00–3.94	−0.130	0.849
Disengagement	586	2.37	0.51	1–4	0.054	0.712
Exhaustion	586	2.68	0.56	1–4	−0.286	0.806
Self-Care Index	585	3.80	0.63	1.35–5.69	−0.047	0.893
Total job performance	584	4.63	0.96	0–6	−1.018	0.871
In-role performance	584	4.58	1.03	0–6	−0.965	0.860
Extra-role performance	584	4.69	1.06	0–6	−0.966	0.754

Note: Scores are presented as mean item scores. In-role performance was calculated from items 2, 3, and 5; extra-role performance was calculated from items 1, 4, and 6; and total job performance was calculated from all six items. Higher scores indicate higher levels of the corresponding construct. The possible score ranges were 0–6 for the UWES-9 and job-performance measures, 1–4 for the OLBI, and 1–6 for the Self-Care Index. Observed ranges refer to the minimum and maximum participant-level mean scores in the study sample. Upper-bound proportions and the proportions of participants with job-performance mean scores ≥ 5.0 are reported in the text. Skewness refers to the adjusted Fisher–Pearson sample coefficient. Sample sizes vary slightly because of missing item responses. UWES-9, Utrecht Work Engagement Scale–9; OLBI, Oldenburg Burnout Inventory; SD, standard deviation. Scale and subscale scores were calculated only when the minimum item-completion requirements described in the Data Analysis subsection were met. The minimum-completion thresholds were operational scoring rules applied during the present reanalysis and were not treated as prospectively prespecified or formally validated instrument-specific missing-item rules. Cronbach’s alpha coefficients were calculated among participants with complete responses to all constituent items of the corresponding scale or subscale. Greek-language UWES item wording had been examined previously, although not as a separate formal validation of the Greek UWES-9 short form; the Greek-language OLBI had also been examined previously. Both were administered in the source survey without further adaptation. The six-item job-performance measure was a previously used Greek-language adaptation of selected items and not a formally validated standalone Greek instrument. The Self-Care Index was a study-developed Greek-language composite, not a validated version of an established international scale. For the Self-Care Index, Cronbach’s alpha was calculated from 515 complete valid item-response records, whereas descriptive statistics were based on the 585 participants meeting the minimum-completion requirement.

### 3.3. Structural Evaluation of the Job-Performance Measure

The dimensional structure of the six-item job-performance adaptation was examined by comparing a one-factor model with a correlated two-factor model representing in-role and extra-role performance. The correlated two-factor model showed improved fit relative to the one-factor model, with χ^2^(8) = 47.26, *p* < 0.001, CFI = 0.996, TLI = 0.992, RMSEA = 0.092 (90% CI: 0.068–0.118), and SRMR = 0.041. Standardized factor loadings ranged from 0.678 to 0.896, and the estimated correlation between the in-role and extra-role factors was 0.833. The fit indices provided a mixed picture: CFI, TLI, and SRMR were favorable, whereas RMSEA suggested residual model misfit. Together with the high factor correlation, these findings provide only partial support for distinguishing the two performance dimensions and indicate substantial shared variance. They should not be interpreted as comprehensive validation of the adaptation or as evidence that the two dimensions are sharply differentiated. Supplementary Table S2 presents the full model-fit indices, standardized item loadings, and estimated factor correlation.

### 3.4. Exploratory Bivariate Associations

Exploratory bivariate analyses indicated that work engagement and Self-Care Index scores were positively associated with each of the three job-performance scores, whereas burnout was inversely associated with them. Several demographic and occupational characteristics also showed nominal associations with individual outcomes. Because these analyses involved multiple exploratory comparisons, were not adjusted for multiplicity, and were not used to select variables for the multivariable models, individual borderline *p*-values are not emphasized here. Full results are provided in Supplementary Table S3. Age and professional experience were strongly correlated (ρ = 0.868, *p* < 0.001); consequently, only age was retained in the common adjustment set.

### 3.5. Multivariable Regression Analyses

All three regression models used the same complete-case sample of 574 participants and the same six-variable adjustment set (Table 3). Twelve of the 586 participants in the source analytical dataset were excluded from the regression analyses because of missing or invalid values required for the common models; the complete derivation is presented in Supplementary Table S5.

For in-role performance, the model accounted for 19.1% of the observed variance (R^2^ = 0.191; adjusted R^2^ = 0.183). Higher work engagement (standardized β = 0.285, *p* < 0.001), higher Self-Care Index scores (β = 0.193, *p* < 0.001), female gender (β = 0.104, *p* = 0.007), and older age (β = 0.100, *p* = 0.013) remained associated with higher self-reported in-role performance after simultaneous adjustment. Burnout did not retain a statistically detectable association with in-role performance after adjustment; managerial responsibility likewise showed no statistically detectable adjusted association with the outcome.

For extra-role performance, the model accounted for 21.2% of the observed variance (R^2^ = 0.212; adjusted R^2^ = 0.204). Work engagement again showed the largest standardized association (β = 0.402, *p* < 0.001). Female gender (β = 0.143, *p* < 0.001) remained positively associated with extra-role performance, while higher Self-Care Index scores showed a comparatively modest positive association (β = 0.100, nominal *p* = 0.018). Burnout did not retain a statistically detectable mutually adjusted association with extra-role performance; age and managerial responsibility likewise showed no statistically detectable adjusted associations with the outcome.

The complementary total-performance model accounted for 23.5% of the observed variance (R^2^ = 0.235; adjusted R^2^ = 0.227). Work engagement (β = 0.376, *p* < 0.001), Self-Care Index scores (β = 0.159, *p* < 0.001), and female gender (β = 0.134, *p* < 0.001) remained positively associated with the total score. Burnout did not retain a statistically detectable mutually adjusted association with total performance; age and managerial responsibility likewise showed no statistically detectable adjusted associations in the primary model.

Variance inflation factors ranged from 1.021 to 2.421 and tolerance values from 0.413 to 0.979. Residual-versus-predicted plots did not indicate marked nonlinearity or a systematic variance pattern in any of the three models, whereas departures from normality in the Q–Q plots were concentrated mainly in the tails. No observation had Cook’s distance greater than 1. The maximum absolute standardized residuals were 6.223, 5.524, and 5.664 for the total, in-role, and extra-role performance models, respectively, and five observations in each model had an absolute standardized residual greater than 3. These observations were retained because no data-quality reason justified their exclusion and none showed substantial overall influence according to Cook’s distance. HC3 sensitivity analyses produced the same overall pattern of statistically detectable and nondetectable associations (Supplementary Table S4). The comparatively modest association between Self-Care Index scores and extra-role performance remained below the nominal 0.05 threshold under HC3 estimation (*p* = 0.033).

Outcome ICCs were low (0.011 for total, 0.007 for in-role, and 0.013 for extra-role performance). In models additionally including hospital fixed effects, the hospital indicators were not jointly associated with any of the three outcomes (all *p* > 0.28), and the principal work-engagement and Self-Care Index associations remained substantively similar (Supplementary Table S6). Age crossed the nominal 0.05 threshold for total performance in this sensitivity model (*p* = 0.042), indicating some inferential sensitivity for this secondary coefficient rather than a new primary finding. These hospital-level analyses should be interpreted as sensitivity checks for between-hospital differences rather than as complete adjustment for all possible within-hospital dependence.

**Table 3:** Multiple linear regression models for in-role, extra-role, and total job performance.

A. In-Role Performance						
Predictor	B	SE	Standardized β	t	*p*	95% CI for B
Constant	1.861	0.538	—	3.462	<0.001	0.805 to 2.917
Gender: female	0.273	0.101	0.104	2.713	0.007	0.075 to 0.471
Age	0.011	0.005	0.100	2.479	0.013	0.002 to 0.020
Managerial responsibility: yes	−0.003	0.132	−0.001	−0.022	0.982	−0.263 to 0.257
Work engagement	0.225	0.046	0.285	4.849	<0.001	0.134 to 0.317
Burnout	0.018	0.122	0.008	0.145	0.885	−0.222 to 0.258
Self-Care Index	0.316	0.070	0.193	4.545	<0.001	0.180 to 0.453
**B. Extra-Role Performance**						
**Predictor**	**B**	**SE**	**Standardized β**	**t**	***p***	**95% CI for B**
Constant	2.150	0.547	—	3.932	<0.001	1.076 to 3.224
Gender: female	0.390	0.103	0.143	3.808	<0.001	0.189 to 0.592
Age	0.004	0.005	0.033	0.826	0.409	−0.005 to 0.013
Managerial responsibility: yes	0.128	0.135	0.039	0.951	0.342	−0.136 to 0.392
Work engagement	0.328	0.047	0.402	6.939	<0.001	0.235 to 0.421
Burnout	0.123	0.124	0.056	0.986	0.325	−0.122 to 0.367
Self-Care Index	0.169	0.071	0.100	2.381	0.018	0.030 to 0.308
**C. Total Job Performance (Complementary Overall Score)**						
**Predictor**	**B**	**SE**	**Standardized β**	**t**	***p***	**95% CI for B**
Constant	2.008	0.488	—	4.119	<0.001	1.051 to 2.966
Gender: female	0.330	0.091	0.134	3.615	<0.001	0.151 to 0.510
Age	0.008	0.004	0.071	1.811	0.071	−0.001 to 0.016
Managerial responsibility: yes	0.063	0.120	0.021	0.527	0.598	−0.172 to 0.299
Work engagement	0.278	0.042	0.376	6.584	<0.001	0.195 to 0.360
Burnout	0.069	0.111	0.035	0.627	0.531	−0.148 to 0.287
Self-Care Index	0.243	0.063	0.159	3.841	<0.001	0.118 to 0.367

Notes: A: F(6, 567) = 22.330; *p* < 0.001; R^2^ = 0.191; adjusted R^2^ = 0.183. B: F(6, 567) = 25.408; *p* < 0.001; R^2^ = 0.212; adjusted R^2^ = 0.204. C: F(6, 567) = 29.025; *p* < 0.001; R^2^ = 0.235; adjusted R^2^ = 0.227. All three models were estimated using the same complete-case sample (*n* = 574) and the same six-predictor adjustment set. Gender was coded as 0 = male and 1 = female, and managerial responsibility was coded as 0 = no and 1 = yes; therefore, male gender and no managerial responsibility served as the respective reference categories. B, unstandardized regression coefficient; SE, standard error; β, standardized regression coefficient; CI, confidence interval. The reported t-tests used 567 residual degrees of freedom. Corresponding HC3 sensitivity estimates are presented in Supplementary Table S4. Coefficient *p*-values are nominal and were not adjusted for multiplicity across the three parallel outcome models.

## 4. Discussion

This secondary analysis examined the mutually adjusted associations of work engagement, burnout, and Self-Care Index scores with self-reported in-role and extra-role performance among nursing personnel from six public hospitals in Northern Greece. Work engagement had the numerically largest standardized coefficient in both the in-role and extra-role models. Higher Self-Care Index scores also showed positive mutually adjusted associations with both outcomes; these associations represent exploratory findings involving the study-developed composite. Burnout was inversely associated with performance in exploratory bivariate analyses but did not retain a statistically detectable association after simultaneous adjustment. The complementary total-performance model showed a similar overall pattern. These findings concern self-reported workforce constructs and should not be interpreted as evidence of causal effects or objectively measured clinical or organizational performance.

The mean work-engagement and burnout scores were close to their scale midpoints, the mean Self-Care Index score was slightly above its response-scale midpoint, and the three performance scores were above the response-scale midpoint. These values should be interpreted descriptively rather than as validated low, moderate, or high levels. Previous healthcare studies have reported variation in work engagement across settings [32], while nursing studies have also reported variation in self-reported job performance [15,16]. Differences across studies may partly reflect variation in measurement instruments, populations, and organizational contexts. Although the comparatively favorable performance scores may serve as workforce indicators, they do not provide evidence of objectively greater productivity, service efficiency, or better patient outcomes. Upper-end concentration in the performance scores also limits interpretation of the association magnitudes. Clustering near the upper response bound may reduce discrimination among participants reporting high performance and compress observable outcome variability. This may attenuate or otherwise alter regression coefficients, including standardized coefficients, and contribute to apparent differences between outcome models. The direction and magnitude of any resulting distortion cannot be determined from the present data. HC3 standard errors do not correct this potential measurement limitation.

The positive association of work engagement with both performance scores is consistent with the broader literature linking engagement with task and contextual performance [10,11,12]. In the present study, the standardized coefficient was numerically larger for extra-role performance (β = 0.402) than for in-role performance (β = 0.285). This comparison is descriptive because no formal test of the difference between these coefficients was performed. Moreover, the CFA provided only partial support for distinguishing the two performance dimensions, given the high factor correlation (r = 0.833) and residual model misfit indicated by RMSEA. The observed coefficient difference therefore does not establish a statistically different association across the two dimensions or a relationship specific to extra-role performance. The relative magnitudes of the standardized coefficients also do not establish the causal importance of engagement or the expected benefit of an intervention targeting it.

Burnout showed inverse exploratory bivariate associations with each performance outcome, consistent with previous evidence linking burnout with impaired occupational functioning among nurses [13]. However, after simultaneous adjustment for work engagement, Self-Care Index scores, gender, age, and managerial responsibility, the burnout coefficients were small and statistically nonsignificant. The resulting positive point estimates should not be interpreted as evidence that greater burnout is associated with better performance. Rather, the bivariate association was attenuated after adjustment for the other variables in the model, and this cross-sectional analysis cannot explain why the coefficient changed.

Higher Self-Care Index scores showed positive mutually adjusted associations with both performance scores. The direction of association is consistent with the earlier Greek critical-care study [15], although the present findings apply specifically to the study-developed 35-item composite. The standardized coefficient was numerically larger for in-role performance (β = 0.193) than for extra-role performance (β = 0.100); this difference is descriptive and was not formally tested. The extra-role association was comparatively modest, with nominal *p*-values of 0.018 in the primary model and 0.033 under HC3 estimation. Because no multiplicity-adjustment strategy was prespecified across the parallel outcome models, this finding should be regarded as hypothesis-generating rather than separately confirmatory. More broadly, the index aggregates heterogeneous behaviors, experiences, and self-perceptions, and the analyses cannot identify which components account for its association with performance. Together with limited construct-validity evidence and possible content overlap with engagement and perceived performance, this supports an exploratory interpretation of all Self-Care Index findings. The results do not demonstrate that increasing the index score or changing any specific self-care behavior would improve job performance.

Female gender was positively associated with the self-reported performance outcomes after adjustment. This finding should not be interpreted as evidence of inherent gender differences in professional ability or used to support gender-based staffing decisions. Unmeasured differences in role allocation, workplace opportunities, assignments, or self-assessment may contribute to the observed association, but the present dataset cannot distinguish among these explanations. Older age was additionally associated with in-role performance. Because age and professional experience were strongly correlated, the finding cannot be attributed specifically to age, accumulated experience, or clinical expertise.

### 4.1. Implications for Healthcare Workforce Management

Engagement-supportive leadership and favorable organizational conditions are plausible areas for prospective management research, based on the present findings and the previous literature [5,20]. Constructive feedback, role clarity, professional-development opportunities, staff participation, mentoring, and recovery opportunities may also warrant evaluation; however, the current study did not directly measure or test these practices, and they should not be presented as interventions shown to improve performance.

Given the exploratory status of the Self-Care Index, the observed associations do not establish that a self-care intervention would improve performance. Future studies should specify the behaviors, experiences, or resources targeted and use measures with appropriate validity evidence. Organizational strategies that facilitate recovery and staff well-being may warrant prospective evaluation, while complementing adequate staffing, manageable workload, and organizational responsibility for working conditions. The observed gender and age associations should not inform demographic-based staffing decisions; instead, they underscore the need to examine whether workplace roles, responsibilities, and developmental opportunities are distributed equitably.

Importantly, the study assessed self-reported job performance rather than objective individual or organizational performance. The study did not measure productivity, costs, resource utilization, service efficiency, absenteeism, turnover, patient safety, patient outcomes, or HRQoL. Future longitudinal and intervention studies should therefore test whether workforce-management strategies addressing engagement, recovery, and staff well-being are associated with independently assessed job performance and objective organizational and patient-level outcomes.

### 4.2. Limitations and Strengths

Several limitations should be considered. First, the source study was cross-sectional; consequently, temporal ordering and causal relationships among engagement, burnout, Self-Care Index scores, and job performance cannot be established. All focal psychosocial variables and performance outcomes were self-reported by the same participants during the same assessment occasion. The observed associations may therefore reflect common-method variance, social-desirability tendencies, consistency motives, or broader response styles. Shared favorable self-appraisal could contribute to higher engagement and Self-Care Index scores as well as higher perceived performance, potentially strengthening some observed associations. The present data cannot distinguish such shared method influences from substantive relationships or quantify their contribution. Neither mutual adjustment nor HC3 or hospital fixed-effects sensitivity analyses remove this limitation. The results therefore concern relationships among self-reported measures and do not establish associations with independently assessed job performance.

Second, the study used a non-probability convenience sample from six public hospitals in Northern Greece. Neither hospitals nor participants were selected using probability-based sampling, and hospital contributions were unequal. Hospital-specific questionnaire-distribution denominators, the total number of eligible personnel approached, and refusal counts were not retained; selection bias therefore cannot be excluded. The source study excluded temporary and short-term contract personnel, further limiting generalizability to those workforce groups. The findings should not be interpreted as nationally representative of Greek nursing personnel.

Third, the original data were collected during the COVID-19 pandemic. Workload, infection-control procedures, organizational disruption, and other pandemic-related workforce conditions may have influenced engagement, burnout, Self-Care Index scores, and perceived performance. The findings therefore reflect a specific historical and organizational context and may not transfer unchanged to contemporary post-pandemic working conditions.

Fourth, the study relied on self-reported performance measured with a six-item Greek-language adaptation that had not previously undergone comprehensive standalone psychometric validation. The structural analysis provided partial support for the correlated two-factor representation, with improved fit relative to the one-factor model; however, the RMSEA indicated residual model misfit and the high factor correlation suggested substantial overlap between the two performance dimensions. Performance scores also showed substantial concentration toward the upper end of the bounded 0–6 response range, particularly for extra-role performance, which may have reduced discrimination among participants reporting high performance. The Self-Care Index was a study-developed multidomain behavioral composite without comprehensive evidence of structural, convergent, discriminant, or criterion validity. The item set combines health-related behaviors with perceived social support, emotional experiences, and work-related beliefs and experiences. Its aggregate score therefore summarizes heterogeneous content, and its associations with engagement and self-reported performance may partly reflect overlapping item content, particularly regarding enjoyment of work and perceived ability to manage occupational challenges. The direction assigned to consumption-related items, including coffee consumption, was an operational scoring choice and was not independently validated in this study. Internal-consistency coefficients in the present sample should therefore not be interpreted as establishing construct validity.

Fifth, the exploratory bivariate analyses involved multiple comparisons, and their *p*-values were not adjusted for multiplicity; isolated nominally significant bivariate findings should therefore be interpreted cautiously. The multivariable analyses used complete cases, although only 12 of 586 participants were excluded from these models. We observed several large standardized residuals and tail departures, but removed no participant solely on statistical diagnostic grounds. Finally, only six hospitals were available for evaluation of hospital-level dependence; the ICC and fixed-effect sensitivity analyses assess sensitivity to between-hospital differences but cannot eliminate all potential within-hospital dependence.

The study also has strengths. It used a relatively large multicentre source dataset and examined both proposed performance scores while explicitly assessing the limitations of their structural distinction. The same adjustment set and complete-case sample were used across models, facilitating descriptive comparison of associations under a consistent specification. Missing-data derivation was reported transparently, and regression diagnostics, HC3 heteroscedasticity-consistent sensitivity analyses, and hospital-level sensitivity analyses were used to assess the stability of the principal findings. These analytical features strengthen the reanalysis’s internal transparency, although they do not overcome the limitations of the original sampling and measurement design.

## 5. Conclusions

In this secondary analysis of a pandemic-era convenience sample from six public hospitals in Northern Greece, work engagement was positively associated with self-reported in-role and extra-role performance and had the numerically largest standardized coefficients in the mutually adjusted models. Higher Self-Care Index scores also showed positive associations, but these findings remain exploratory and apply specifically to the study-developed composite, whose construct validity has not been established. Burnout did not retain a statistically detectable association after simultaneous adjustment. Interpretation is constrained by partial support for distinguishing the performance dimensions, upper-end score concentration, and reliance on simultaneous self-reports. The findings identify workforce associations for further investigation but do not establish causal effects or effective management interventions. Longitudinal and intervention studies should use validated measures, independently assessed performance, and objective organizational and patient outcomes to evaluate whether specific workforce-management strategies produce measurable benefits.

## Data Availability

The data supporting the findings of this secondary analysis are held by the research team and may be available from the corresponding author on reasonable request, subject to the conditions of the original ethics approval, participant consent, and applicable institutional data-governance requirements. The dataset has not been deposited in a public repository.

## Supplementary Materials

The following supporting information can be downloaded at: https://www.mdpi.com/article/10.3390/healthcare14203460/s1, Table S1: Distribution of returned questionnaires included as valid responses across the six participating hospitals; Table S2: Structural evaluation of the six-item job-performance measure; Table S3: Exploratory bivariate associations of participant characteristics and study variables with self-reported job-performance outcomes; Table S4: Sensitivity analyses of the associations with total, in-role, and extra-role job performance using HC3 heteroscedasticity-consistent standard errors; Table S5: Variable-level missingness and derivation of the regression sample; Table S6: Hospital fixed-effects sensitivity analyses of the associations with total, in-role, and extra-role job performance; Table S7: STROBE checklist for cross-sectional studies; Table S8: Item-content descriptors, questionnaire numbering, dataset mapping, and operational scoring of the 35-item Self-Care Index; Table S9: Item-content descriptors, questionnaire numbering, dataset mapping, and operational scoring of the six-item self-reported job-performance adaptation.

## Abbreviations

HRQoL	health-related quality of life
JP	job performance
OLBI	Oldenburg Burnout Inventory
UWES	Utrecht Work Engagement Scale
VIFs	variance inflation factors
