# Supplementary material for: Work Engagement, Burnout, and Self-Care Index Scores in Relation to Self-Reported In-Role and Extra-Role Job Performance Among Nursing Personnel in Six Public Hospitals in Northern Greece: A Secondary Analysis of a Multicentre Cross-Sectional Survey
Source: Healthcare (Basel). 2026 Oct 15;14(20):3460. doi: 10.3390/healthcare14203460 (PMC13704241; doi:10.3390/healthcare14203460)
Supplement: Supplementary file 1 [file healthcare-14-03460-s001.zip › healthcare-4578457-supplementary.pdf]

Supplementary Table S1. Distribution of returned questionnaires included as valid responses across the six participating hospitals.

Participating hospital	Hospital level	Questionnaires returned and included, n	Percentage of included sample, %	Questionnaires distributed, n	Hospital-specific return rate
Papageorgiou General Hospital, Thessaloniki	Tertiary	246	42.0	Not retained	Not calculable
AHEPA University General Hospital, Thessaloniki	Tertiary	127	21.7	Not retained	Not calculable
Agios Dimitrios General Hospital, Thessaloniki	Secondary	68	11.6	Not retained	Not calculable
G. Gennimatas General Hospital, Thessaloniki	Secondary	57	9.7	Not retained	Not calculable
Grevena General Hospital	Secondary	49	8.4	Not retained	Not calculable
Bodosakeio General Hospital, Ptolemaida	Secondary	39	6.7	Not retained	Not calculable
Total	—	586	100.0	817	71.7% overall questionnaire return rate

Notes: The table reports the hospital affiliation of the 586 returned questionnaires included in the study. A total of 817 questionnaires were distributed across the six hospitals. However, the number distributed at each hospital, the total number of eligible personnel approached, and the number declining participation were not retained. Consequently, hospital-specific questionnaire return rates could not be calculated. The overall 71.7% figure represents the proportion of distributed questionnaires that were returned and included and should not be interpreted as a probability-sample or population-based response rate. Percentages may not sum to exactly 100.0% because of rounding.

Supplementary Table S2. Structural evaluation of the six-item job-performance measure.

Model	χ^2	df	p	CFI	TLI	RMSEA (90% CI)	SRMR
One-factor model	114.21	9	<0.001	0.989	0.982	0.142 (0.120–0.166)	0.063
Correlated two-factor model	47.26	8	<0.001	0.996	0.992	0.092 (0.068–0.118)	0.041

Item	Assigned factor	Standardized loading
JP1	Extra-role performance	0.792

Item	Assigned factor	Standardized loading
JP2	In-role performance	0.843
JP3	In-role performance	0.896
JP4	Extra-role performance	0.889
JP5	In-role performance	0.853
JP6	Extra-role performance	0.678
**Factor correlation**	**In-role ↔ Extra-role**	**0.833**

Note: Confirmatory factor analysis was conducted on the six ordered-response job-performance items using a diagonally weighted least-squares approach based on polychoric correlations. The CFA analytic sample comprised 579 participants with complete responses to all six job-performance items; seven participants with incomplete item-level data were excluded, and no imputation was performed. Analyses were conducted in JASP (Version 0.17) using the SEM/CFA module (lavaan engine). The correlated two-factor model specified JP2, JP3, and JP5 as indicators of in-role performance and JP1, JP4, and JP6 as indicators of extra-role performance. The fit indices provide only partial support for distinguishing in-role and extra-role performance. Improved fit relative to the one-factor model should be interpreted alongside residual RMSEA misfit and the high factor correlation ($r = 0.833$), which indicates substantial overlap between the dimensions. These sample-specific findings do not constitute comprehensive psychometric validation of the adaptation. CFI, comparative fit index; TLI, Tucker–Lewis index; RMSEA, root mean square error of approximation; CI, confidence interval; SRMR, standardized root mean square residual.

Supplementary Table S3. Exploratory bivariate associations of participant characteristics and study variables with self-reported job-performance outcomes.

Independent variable	Total JP, statistic	p-value	n	In-role JP, statistic	p-value	n	Extra-role JP, statistic	p-value	n
Gender[a]	U = 20,585.0	<0.001	584	U = 21,350.5	0.004	584	U = 20,482.5	<0.001	584
Age[b]	$\rho = 0.103$	0.013	578	$\rho = 0.103$	0.013	578	$\rho = 0.085$	0.042	578
Marital status[a]	U = 33,726.0	0.014	582	U = 34,770.5	0.054	582	U = 33,936.5	0.018	582
Professional role[a]	U = 33,476.5	0.899	584	U = 35,363.5	0.236	584	U = 32,402.5	0.637	584
Educational level[c]	H = 0.891	0.640	584	H = 4.709	0.095	584	H = 0.039	0.981	584
Professional experience[b]	$\rho = 0.126$	0.002	576	$\rho = 0.142$	<0.001	576	$\rho = 0.088$	0.035	576
Clinical department[a]	U = 37,609.0	0.338	538	U = 37,488.5	0.371	538	U = 37,305.5	0.429	538
Managerial responsibility[a]	U = 12,707.0	<0.001	584	U = 13,559.0	0.001	584	U = 13,041.0	<0.001	584
Hospital level[a]	U = 37,528.0	0.331	584	U = 37,185.0	0.248	584	U = 37,684.0	0.370	584
UWES-9[b]	$\rho = 0.443$	<0.001	581	$\rho = 0.370$	<0.001	581	$\rho = 0.428$	<0.001	581
OLBI[b]	$\rho = -0.305$	<0.001	584	$\rho = -0.261$	<0.001	584	$\rho = -0.285$	<0.001	584

Self-Care Index[b]	ϱ = 0.314	<0.001	583	ϱ = 0.307	<0.001	583	ϱ = 0.273	<0.001	583

Notes: [a] Mann–Whitney U test; [b] Spearman's rank correlation coefficient; [c] Kruskal–Wallis H test. All tests were two-sided. Valid sample sizes vary because of missing or invalid data and, for the clinical department, because only participants who could be classified into one of two analytic clinical-department groups were included. For continuous variables, positive and negative ϱ values indicate positive and inverse associations, respectively. Exact p-values are reported to three decimal places; values below 0.001 are reported as $p < 0.001$. For marital status, we compared married participants with those who were single, divorced, or widowed. These analyses were exploratory. P-values were not adjusted for multiple comparisons and were not used to determine inclusion in the multivariable models.

Abbreviations: JP, job performance; UWES-9, Utrecht Work Engagement Scale–9; OLBI, Oldenburg Burnout Inventory; U, Mann–Whitney U statistic; H, Kruskal–Wallis statistic; ϱ, Spearman's rank correlation coefficient.

Supplementary Table S4. Sensitivity analyses of the associations with total, in-role, and extra-role job performance using HC3 heteroscedasticity-consistent standard errors.

Total job performance

Predictor	B	HC3 SE	HC3 95% CI	t	p
Intercept	2.008	0.583	0.864 to 3.153	3.446	0.001
Female gender	0.330	0.093	0.147 to 0.514	3.543	<0.001
Age	0.008	0.005	−0.001 to 0.016	1.643	0.101
Managerial responsibility	0.063	0.106	−0.144 to 0.271	0.599	0.550
Work engagement	0.278	0.043	0.194 to 0.361	6.503	<0.001
Burnout	0.069	0.111	−0.148 to 0.287	0.627	0.531
Self-Care Index	0.243	0.076	0.094 to 0.391	3.208	0.001

In-role job performance

Predictor	B	HC3 SE	HC3 95% CI	t	p
Intercept	1.861	0.633	0.618 to 3.104	2.940	0.003
Female gender	0.273	0.100	0.076 to 0.471	2.726	0.007
Age	0.011	0.005	0.002 to 0.021	2.277	0.023
Managerial responsibility	−0.003	0.126	−0.251 to 0.245	−0.024	0.981
Work engagement	0.225	0.047	0.132 to 0.318	4.754	<0.001
Burnout	0.018	0.122	−0.223 to 0.258	0.145	0.885
Self-Care Index	0.316	0.087	0.146 to 0.487	3.644	<0.001

Extra-role job performance

Predictor	B	HC3 SE	HC3 95% CI	t	p

Intercept	2.150	0.617	0.937 to 3.363	3.483	0.001
Female gender	0.390	0.111	0.173 to 0.608	3.523	<0.001
Age	0.004	0.005	−0.006 to 0.014	0.753	0.452
Managerial responsibility	0.128	0.108	−0.085 to 0.340	1.182	0.238
Work engagement	0.328	0.047	0.235 to 0.421	6.932	<0.001
Burnout	0.123	0.125	−0.123 to 0.368	0.979	0.328
Self-Care Index	0.169	0.079	0.014 to 0.323	2.140	0.033

Notes: All three models were estimated using the same complete-case sample (n = 574) and the same six predictors as the primary OLS models. HC3, heteroscedasticity-consistent covariance matrix estimator type 3; B, unstandardized regression coefficient; SE, standard error; CI, confidence interval. The reported t-tests used 567 residual degrees of freedom. Reference categories were male gender and no managerial responsibility.

Supplementary Table S5. Variable-level missingness and derivation of the regression sample.

Variable or analytic requirement	Full sample	Valid n	Missing/invalid /unclassifiable n	Explanation
Gender	586	586	0	—
Age	586	580	6	Five missing; one value of 0 treated as invalid
Marital status	586	584	2	Missing responses
Professional role	586	586	0	—
Educational level	586	586	0	—
Professional experience	586	577	9	Missing responses
Hospital level	586	586	0	—
Managerial responsibility	586	586	0	—
Clinical department recorded	586	559	27	No department recorded
Clinical department classifiable	586	538	48	27 missing and 21 unclassifiable placements
UWES-9 total score	586	583	3	No UWES-9 items completed
OLBI total score	586	586	0	All met the completion threshold
Self-Care Index	586	585	1	Only 22 of 35 items completed

Total job performance	586	584	2	No job-performance items completed
In-role performance	586	584	2	No job-performance items completed
Extra-role performance	586	584	2	No job-performance items completed
Complete data for all regression variables	586	574	12	Six age exclusions, three UWES-9 exclusions, two job-performance exclusions, and one Self-Care Index exclusion

Note: Variable-level missingness counts should not be added across variables because missingness may overlap across measures. For the regression models, 12 participants were excluded in four mutually exclusive categories: six because of missing or invalid age values, three because of unavailable UWES-9 scores, two because of unavailable job-performance scores, and one because of an unavailable Self-Care Index score. The 21 unclassifiable clinical-department placements were recorded responses and are not missing data in the strict sense. Scale-score validity in this table reflects the operational minimum-completion thresholds applied during the present reanalysis, as described in Section 2.6 of the main manuscript. For the Self-Care Index, 97 item responses were blank and six recorded values fell outside the permitted 1–6 range; the latter were treated as missing before scoring. After screening, 515 participants had 35 valid item responses, 57 had 34, seven had 33, five had 32, one had 31, and one had 22. The participant with 22 valid responses did not meet the minimum-completion requirement, leaving 585 participants with a valid Self-Care Index score. Cronbach's alpha was calculated using the 515 participants with complete valid responses to all 35 items.

Supplementary Table S6. Hospital fixed-effects sensitivity analyses of the associations with total, in-role, and extra-role job performance.

Predictor	Total performance B (95% CI)	p	In-role performance B (95% CI)	p	Extra-role performance B (95% CI)	p
Female gender	0.331 (0.151 to 0.511)	<0.001	0.272 (0.074 to 0.471)	0.007	0.392 (0.190 to 0.594)	<0.001
Age	0.009 (0.0003 to 0.017)	0.042	0.012 (0.002 to 0.021)	0.014	0.006 (−0.003 to 0.015)	0.219
Managerial responsibility	0.038 (−0.201 to 0.278)	0.752	−0.002 (−0.266 to 0.262)	0.989	0.078 (−0.190 to 0.346)	0.568
Work engagement	0.281 (0.197 to 0.364)	<0.001	0.225 (0.133 to 0.318)	<0.001	0.334 (0.240 to 0.428)	<0.001
Burnout	0.091 (−0.128 to 0.309)	0.416	0.036 (−0.205 to 0.277)	0.771	0.146 (−0.099 to 0.391)	0.241

Self-Care Index	0.247 (0.121 to 0.372)	<0.001	0.326 (0.188 to 0.465)	<0.001	0.167 (0.027 to 0.307)	0.020
Joint test of hospital indicators	F(5, 562) = 1.060	0.381	F(5, 562) = 1.059	0.382	F(5, 562) = 1.241	0.288

Model R^2: total performance, 0.242; in-role performance, 0.199; extra-role performance, 0.221.
Adjusted R^2: total performance, 0.227; in-role performance, 0.183; extra-role performance, 0.205.

Note: The hospital fixed-effects models used the same complete-case sample of 574 participants as the primary models. Each model included the six primary predictors and five hospital indicator variables. These analyses assessed sensitivity to between-hospital differences and should not be interpreted as fully accounting for all possible within-hospital dependence.

Supplementary Table S7. STROBE checklist for cross-sectional studies

Section/topic	Item no.	STROBE recommendation	Location in manuscript
Title and abstract	1	(a) Indicate the study's design with a commonly used term in the title or the abstract.	Title; Abstract (Methods)
		(b) Provide in the abstract an informative and balanced summary of what was done and what was found.	Abstract
Introduction—Background/rationale	2	Explain the scientific background and rationale for the investigation being reported.	Section 1, Introduction, paragraphs 1–4
Introduction—Objectives	3	State specific objectives, including any prespecified hypotheses.	Section 1, Introduction, final paragraph
Methods—Study design	4	Present key elements of study design early in the paper.	Section 2.1, Study Design, Data Source, and Setting
Methods—Setting	5	Describe the setting, locations, and relevant dates, including periods of recruitment, exposure, follow-up, and data collection.	Sections 2.1–2.2; Supplementary Table S1
Methods—Participants	6	Give the eligibility criteria and the sources and methods of selection of participants.	Section 2.2, Data Collection
Methods—Variables	7	Clearly define all outcomes, exposures, predictors, potential confounders, and effect modifiers. Give diagnostic criteria, if applicable.	Sections 2.3–2.4 and 2.6
Methods—Data sources/measurement	8	For each variable of interest, give sources of data and details of methods of assessment. Describe comparability of assessment methods if there is more than one group.	Sections 2.2–2.4.6 and 2.6; Supplementary Tables S8 and S9.
Methods—Bias	9	Describe any efforts to address potential sources of bias.	Sections 2.1–2.2 and 2.6; Section 4.2

Methods—Study size	10	Explain how the study size was arrived at.	Section 2.3, Study Size and Analytical Sample
Methods—Quantitative variables	11	Explain how quantitative variables were handled in the analyses. If applicable, describe which groupings were chosen and why.	Sections 2.4.1–2.4.5 and 2.6; Table 2 and its notes; Supplementary Tables S8 and S9.
Methods—Statistical methods	12(a)	Describe all statistical methods, including those used to control for confounding.	Section 2.6, Data Analysis
	12(b)	Describe any methods used to examine subgroups and interactions.	Exploratory bivariate group comparisons: Sections 2.6 and 3.4; Supplementary Table S3. No interaction terms or stratified regression models were fitted.
	12(c)	Explain how missing data were addressed.	Sections 2.6, 3.1, and 3.5; Supplementary Tables S2 and S5.
	12(d)	If applicable, describe analytical methods taking account of sampling strategy.	Section 2.6; Probability-sampling weights were not applicable. We evaluated hospital-level sensitivity using ICC estimates and hospital fixed-effects models.
	12(e)	Describe any sensitivity analyses.	Section 2.6; Section 3.5; Supplementary Tables S4 and S6
Results—Participants	13(a)	Report numbers of individuals at each stage of the study—for example, potentially eligible, examined for eligibility, confirmed eligible, included, and analysed.	Sections 2.2–2.3, 2.6, 3.1, and 3.5; Supplementary Tables S1, S2, and S5.
	13(b)	Give reasons for non-participation at each stage.	Sections 2.2, 4.2; Section 3.5 and S5

	13(c)	Consider use of a flow diagram.	No flow diagram was included. Participant flow and analysis-specific sample sizes are reported in Sections 2.2–2.3, 2.6, 3.1, and 3.5; Supplementary Tables S1, S2, and S5.
Results—Descriptive data	14(a)	Give characteristics of study participants and information on exposures and potential confounders.	Section 3.1; Tables 1–2; Supplementary Table S1
	14(b)	Indicate the number of participants with missing data for each variable of interest.	Section 2.6; Sections 3.1–3.2; Tables 1–2; Supplementary Tables S2 and S5.
	14(c)	Summarise follow-up time (for cohort studies).	Not applicable—cross-sectional study
Results—Outcome data	15	Report numbers of outcome events or summary measures.	Section 3.2; Table 2
Results—Main results	16(a)	Give unadjusted estimates and, if applicable, confounder-adjusted estimates and their precision. Make clear which confounders were adjusted for and why they were included.	Sections 2.6 and 3.4–3.5; Table 3; Supplementary Table S3.
	16(b)	Report category boundaries when continuous variables were categorised.	Sections 2.4.1 and 2.6; Table 1 and Supplementary Table S3 notes. The principal continuous predictors were analyzed as continuous scores.
	16(c)	If relevant, consider translating estimates of relative risk into absolute risk for a meaningful time period.	Not applicable—continuous outcomes and linear regression; no risk estimates

Results—Other analyses	17	Report other analyses performed, such as subgroup, interaction, and sensitivity analyses.	Sections 3.3 and 3.5; S2, S4 and S6
Discussion—Key results	18	Summarise key results with reference to study objectives.	Section 4, Discussion, opening paragraph; Section 5, Conclusions
Discussion—Limitations	19	Discuss limitations, considering sources of potential bias or imprecision. Discuss both direction and magnitude of any potential bias.	Section 4.2, Limitations and Strengths
Discussion—Interpretation	20	Give a cautious overall interpretation considering objectives, limitations, multiplicity of analyses, results from similar studies, and other relevant evidence.	Sections 4–4.2; Section 5
Discussion—Generalisability	21	Discuss the generalisability (external validity) of the study results.	Section 4.2, Limitations and Strengths
Other information—Funding	22	Give the source of funding and the role of the funders for the present study and, if applicable, for the original study on which the present article is based.	Funding statement: no external funding

Supplementary Table S8. Item-content descriptors, questionnaire numbering, dataset mapping, and operational scoring of the 35-item Self-Care Index.

SCI item	Questionnaire item	Dataset variable — Excel column	Brief content descriptor	Scoring
1	48	SELF CARE (1) — AW	Breakfast consumption	x
2	49	SELF CARE (2) — AX	Fast-food consumption	7 − x
3	50	SELF CARE (3) — AY	Hydration	x
4	51	SELF CARE (4) — AZ	Daily exercise duration	x
5	52	SELF CARE (5) — BA	Nightly sleep duration	x

6	53	SELF CARE (6) — BB	Relaxation activities	x
7	54	SELF CARE (7) — BC	Alcohol consumption	7 − x
8	55	SELF CARE (8) — BD	Tobacco smoking	7 − x
9	56	SELF CARE (9) — BE	Coffee consumption	7 − x
10	57	SELF CARE (10) — BF	Leisure interests	x
11	58	SELF CARE (11) — BG	Meditation and reflection	x
12	59	SELF CARE (12) — BH	Perceived interpersonal understanding and recognition	x
13	60	SELF CARE (13) — BI	Life meaning during adversity	x
14	61	SELF CARE (14) — BJ	Positive future expectations	x
15	62	SELF CARE (15) — BK	Supportive self-talk	x
16	63	SELF CARE (16) — BL	Self-kindness	x
17	64	SELF CARE (17) — BM	Spontaneous emotional expression	x
18	65	SELF CARE (18) — BN	Constructive anger expression	x
19	66	SELF CARE (19) — BO	Personal aspirations and goals	x
20	67	SELF CARE (20) — BP	Limits on additional responsibilities under stress	x
21	68	SELF CARE (21) — BQ	Receptiveness to receiving as well as giving	x

22	69	SELF CARE (22) — BR	Work interest and enjoyment	x
23	70	SELF CARE (23) — BS	Empathy in clinical and collegial interactions	x
24	71	SELF CARE (24) — BT	Perceived capacity to address workplace challenges	x
25	72	SELF CARE (25) — BU	Boundary setting	x
26	73	SELF CARE (26) — BV	Limiting performance comparisons with colleagues	x
27	74	SELF CARE (27) — BW	Seeking social support	x
28	75	SELF CARE (28) — BX	Disengagement from daily pressures	x
29	76	SELF CARE (29) — BY	Holidays and restorative breaks	x
30	77	SELF CARE (30) — BZ	Dependable interpersonal support	x
31	78	SELF CARE 31) — CA	Family support	x
32	79	SELF CARE (32) — CB	Positive social contact	x
33	80	SELF CARE (33) — CC	Assertiveness in declining requests	x
34	81	SELF CARE (34) — CD	Work–life balance	x
35	82	SELF CARE (35) — CE	Perceived ability to seek help	x

Note: SCI, Self-Care Index. The brief content descriptors were prepared by the authors for methodological documentation. They are not verbatim questionnaire items, item translations, or wording intended for administration. Item numbering and dataset mapping were verified against the archived final Greek questionnaire and the source dataset. "SCI item" denotes the sequential position within the 35-item index; "Questionnaire item" denotes the numbering in the complete survey. Excel column references correspond to the worksheet "ΔΕΔΟΜΕΝΑ". The variable header "SELF CARE 31)" is retained as recorded.

Response coding and recall period: Responses were coded as 1 = never, 2 = rarely, 3 = sometimes, 4 = often, 5 = regularly, and 6 = always. The original ordering of response categories was retained. Instructions referred to participants' current self-care practices without specifying a fixed recall interval.

Operational scoring: In the scoring column, x denotes the original valid response. Items 2, 7, 8, and 9 were reverse-coded as 7 − x; the remaining 31 items retained their original coding. Blank responses and values outside the permitted 1–6 range were treated as missing before scoring. The overall score was calculated as the arithmetic mean of valid, directionally coded responses when at least 28 of the 35 items were available, using the number of valid responses as the participant-specific denominator. Otherwise, the score was coded as missing. No missing responses were imputed, and no domain-specific scores were calculated. The possible score range was 1–6. Higher scores indicate a more favorable response pattern under the operational scoring scheme. The reverse-coding decisions and minimum-completion requirement were operational choices for the present reanalysis and were not independently validated instrument-specific rules.

Source documentation: The administered item set originated from the survey reported by Kostelidou [21]. Its documented conceptual sources and the limitations of the available development records are described in Section 2.4.4 of the main article. Reference numbers correspond to the main article. The index remains an exploratory multidomain composite without comprehensive psychometric validation.

Supplementary Table S9. Item-content descriptors, questionnaire numbering, dataset mapping, and operational scoring of the six-item self-reported job-performance adaptation.

**JP item**	**Questionnaire item**	**Dataset variable — Excel column**	**Brief content descriptor**	**Analytic score allocation**	**Scoring**
1	42	JP (1) — AQ	Assistance to colleagues returning from absence	Extra-role performance	x
2	43	JP (2) — AR	Attainment of job objectives	In-role performance	x
3	44	JP (3) — AS	Fulfilment of performance standards	In-role performance	x
4	45	JP (4) — AT	Assistance to colleagues facing workload or other difficulties	Extra-role performance	x
5	46	JP (5) — AU	Fulfilment of job requirements	In-role performance	x
6	47	JP (6) — AV	Voluntary contribution beyond assigned duties	Extra-role performance	x

Note: JP, job performance. The brief content descriptors were prepared by the authors for methodological documentation. They are not verbatim questionnaire items, item translations, or wording intended for administration. Item numbering and dataset mapping were verified against the archived final Greek questionnaire and the source dataset. "JP item" denotes the sequential position within the six-item adaptation; "Questionnaire item" denotes the numbering in the complete survey. Excel column references correspond to the worksheet "ΔΕΔΟΜΕΝΑ".

Response coding and recall period: Participants were instructed to refer to the preceding four months. Responses used a seven-category scale ranging from 0, indicating that the statement did not characterize the respondent at all, to 6, indicating that it characterized the respondent completely. Zero was a valid response.

Operational scoring: In the scoring column, x denotes the original valid response. All six items retained their original coding; no items were reverse-coded. Blank responses and values outside the permitted 0–6 range were treated as missing before scoring.

In-role performance was calculated as the arithmetic mean of valid responses to items 2, 3, and 5, provided that at least two of the three items were available. Extra-role performance was calculated as the arithmetic mean of valid responses to items 1, 4, and 6, provided that at least two of the three items were available. The complementary total score was calculated directly as the arithmetic mean of valid responses across all six items, provided that at least five items were available.

For each score, the denominator was the number of valid responses to its constituent items. Scores were coded as missing when the corresponding minimum-completion requirement was not met. No missing responses were imputed. All three scores had a possible range of 0–6, with higher values indicating higher self-reported performance. The total score was not calculated by averaging the two domain means, because that approach can produce a different result when an item is missing. The minimum-completion requirements were operational rules for the present reanalysis and were not prospectively prespecified or independently validated instrument-specific rules.

Source documentation: The administered Greek-language adaptation was documented in the original survey [21], which attributed the adaptation to Karagkounis [31], drawing on Goodman and Svyantek [30]. Reference numbers correspond to the main article. This table documents the six-item version used in the study and does not establish equivalence to the original instrument or validation of a standalone Greek short form. The total score was retained as a complementary summary and was not interpreted as a psychometrically distinct third dimension.
